# Relationship between Change in Bone Mineral Density of Lumbar Spine and Risk of New Vertebral and Nonvertebral Fractures: A Meta‐Analysis

**DOI:** 10.1111/os.13184

**Published:** 2022-01-04

**Authors:** Liang Chen, Xiao‐ping Liu, Bo Zhou, Tong‐ya Guo, Feng Yuan, Mohamed E A Abdelrahim, Zhen‐huan Jiang

**Affiliations:** ^1^ Department of Orthopaedics Yixing People's Hospital Yixing China; ^2^ Department of Orthopaedics Suzhou Kowloon Hospital Shanghai Jiao Tong University School of Medicine Suzhou China; ^3^ Department of Bone and Joint Surgery Xuzhou Central Hospital Xuzhou China; ^4^ Department of Orthopaedic Surgery Shanghai Jiao Tong University Affiliated Sixth People's Hospital Shanghai 200233 China; ^5^ Clinical Pharmacy Department Faculty of Pharmacy, Beni‐Suef University Beni‐Suef Egypt

**Keywords:** Bone mineral density, Lumbar spine, Nonvertebral fracture, Postmenopausal females, Vertebral fracture

## Abstract

Studies have shown that the change in lumbar spine bone mineral density with different osteoporosis drugs had a beneficial effect on the frequency of new vertebral and nonvertebral fractures in postmenopausal females, but their results were conflicting. This meta‐analysis was performed to evaluate this relationship. A systematic literature search up to May 2020 was performed and 20 studies with 73,390 postmenopausal females were included; of them, a total of 41,980 were treated with osteoporosis drugs and 31,410 with placebo. They reported relationships between the change in lumbar spine bone mineral density and the frequency of new vertebral and nonvertebral fractures in postmenopausal females. Odds ratio (*OR*) with 95% confidence intervals (*CI*s) was calculated comparing the osteoporosis drugs to placebo effect on the frequency of new vertebral and nonvertebral fractures in postmenopausal females using the dichotomous method with a random or fixed‐effect model. Treatment with osteoporosis drugs had significantly lower frequency of new vertebral fractures (*OR*, 0.53; 95% *CI*, 0.45–0.63, *P* < 0.001) and nonvertebral fractures (*OR*, 0.82; 95% *CI*, 0.78–0.87, *P* < 0.001) compared to placebo in postmenopausal females. Treatment with osteoporosis drugs had a significantly lower frequency of new vertebral and nonvertebral fractures compared to placebo in postmenopausal females. This relationship forces us to recommend osteoporosis drugs in postmenopausal females to avoid any possible new fractures. A cost‐effective study is recommended.

## Graphical abstract

Description of the meta‐analysis procedure.

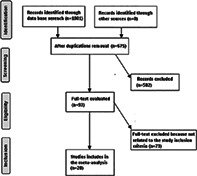

## Introduction

The development of models to forecast fracture results has been discussed in several meetings and workshops, e.g. at the 2015 Food and Drug Administration Scientific Workshop and Osteoporosis Drug Development. The relations between variation in bone mineral density and fracture decrease was highly discussed on the agenda. Investigation of clinical studies with strontium ranelate reported no relationship between lumbar bone mineral density variation and the frequency of vertebral fractures and nonvertebral fractures^1^.

It was also reported that when interpreting the association between the increase in bone mineral density with vertebral fractures and nonvertebral, risk decrease by strontium ranelate treatment. It is essential to think through what part of the variations in bone mineral density by strontium ranelate treatment was caused by the higher atomic number of strontium (*Z* = 38) than the atomic number of calcium (*Z* = 20)^2^. The Food and Drug Administration and European Medicines Agency asked for evidence of fracture decrease efficiency in osteoporosis drug development and have uncertainties about the use of bone mineral density alone for fracture in randomized clinical trials^3^, ^4^. When bone mineral density is measured by dual‐energy X‐ray absorptiometry, strontium atoms in the bone reduce in X‐rays more than calcium, causing over the assessment of the bone mineral density^5^. However, a larger increase in lumbar spine bone mineral density by alendronate treatments revealed a significant association with a lower risk of vertebral fracture^6^. A systematic review examined the association between the relative risks of vertebral fractures and nonvertebral fractures and intensifies the bone mineral density since a larger increase in bone mineral density is inclined to have greater anti‐fracture effectiveness^7^. In these studies, however, the effects of other factors on the relationship were not measured. The changes in the ratio of subjects with predominant fracture between studies were masked in these studies.

A former meta‐analysis of 11 cohort studies, in which osteoporotic fracture history and follow‐up of fracture for individual subjects were performed, showed an association between past fractures and successive fractures^8^. The diagnosis guidelines for osteoporosis^9^ as well as the inclusion criteria for randomized clinical trials of osteoporosis drugs describe predominant osteoporotic fracture, including vertebral fractures and nonvertebral fractures, as a significant diagnostic criterion of osteoporosis. Previously, a study examined the relationship between the frequency of vertebral fractures and nonvertebral fractures in the placebo group and numerous demographic factors at baseline^10^.

Outcomes of this study showed that the proportion of subjects with predominant vertebral fractures and nonvertebral fractures had anassociation with the frequency of fracture, but the baseline bone mineral density T‐score did not demonstrate a significant relationship with the frequency of vertebral fractures and nonvertebral fractures^10^. These outcomes showed that baseline bone mineral density T‐scores do not forecast the frequency of vertebral fractures and nonvertebral fractures in the 3‐year study period and recommend the need to assess the relationship between change in lumbar spine bone mineral density and the frequency of vertebral fractures and nonvertebral fractures.

Previous studies of osteoporosis drugs and a systematic review reported that a larger increase in bone mineral density tended to have greater anti‐fracture efficacy^6^, ^7^. Although the change in lumbar spine bone mineral density showed a significant correlation with the incidence of new vertebral fractures and nonvertebral fractures, regardless of the adjustment with the proportion, bone mineral density showed a significant correlation with the incidence of new vertebral fractures and nonvertebral fractures in both the higher and lower tertile group without the adjustment with the proportion of subjects with prevalent vertebral fractures and nonvertebral fractures^11^, ^12^, ^13^, ^14^, ^15^, ^16^, ^17^, ^18^, ^19^, ^20^, ^21^, ^22^, ^23^, ^24^, ^25^, ^26^, ^27^, ^28^, ^29^, ^30^. Therefore, we suggest that the main factor leading to a model fitting in the meta‐analysis study was the difference in the risk of new vertebral fractures and nonvertebral fractures among the study populations with different prevalence of vertebral fractures and nonvertebral fractures.

This indicates that the correlation between the change in bone mineral density and the incidence of new vertebral fractures and nonvertebral fractures is different between the study populations with a high and low prevalence of vertebral fractures and nonvertebral fractures; the higher prevalence of vertebral fractures and nonvertebral fractures the study group has, the greater the effect of the increase in lumbar spine bone mineral density on the prevention of new vertebral fractures and nonvertebral fractures observed. The degree of prevalence of vertebral fractures and nonvertebral fractures in the population should be considered when the association between change in lumbar spine bone mineral density and incidence of vertebral fractures and nonvertebral fractures is examined. From all this, it is obvious that studies have shown that the change in lumbar spine bone mineral density with different osteoporosis drugs had a beneficial effect on the frequency of new vertebral fractures and nonvertebral fractures in postmenopausal females, but their results were conflicting^11^, ^12^, ^13^, ^14^, ^15^, ^16^, ^17^, ^18^, ^19^, ^20^, ^21^, ^22^, ^23^, ^24^, ^25^, ^26^, ^27^, ^28^, ^29^, ^30^.

The present meta‐analysis study aimed to examine the relationship between the change in lumbar spine bone mineral density and the frequency of new vertebral fractures and nonvertebral fractures in postmenopausal females.

## Methods

The study performed here followed the meta‐analysis of studies in the epidemiology statement^31^, which was conducted following an established protocol as shown in Table S1 for PRISMA checklist as a basis for reporting systematic reviews objectives and evaluating interventions.

### Study Selection

Studies included were retrospective or randomized clinical trials evaluating the relationship between the change in lumbar spine bone mineral density and the frequency of new vertebral fractures and nonvertebral fractures in postmenopausal females.

**Fig. 1:**
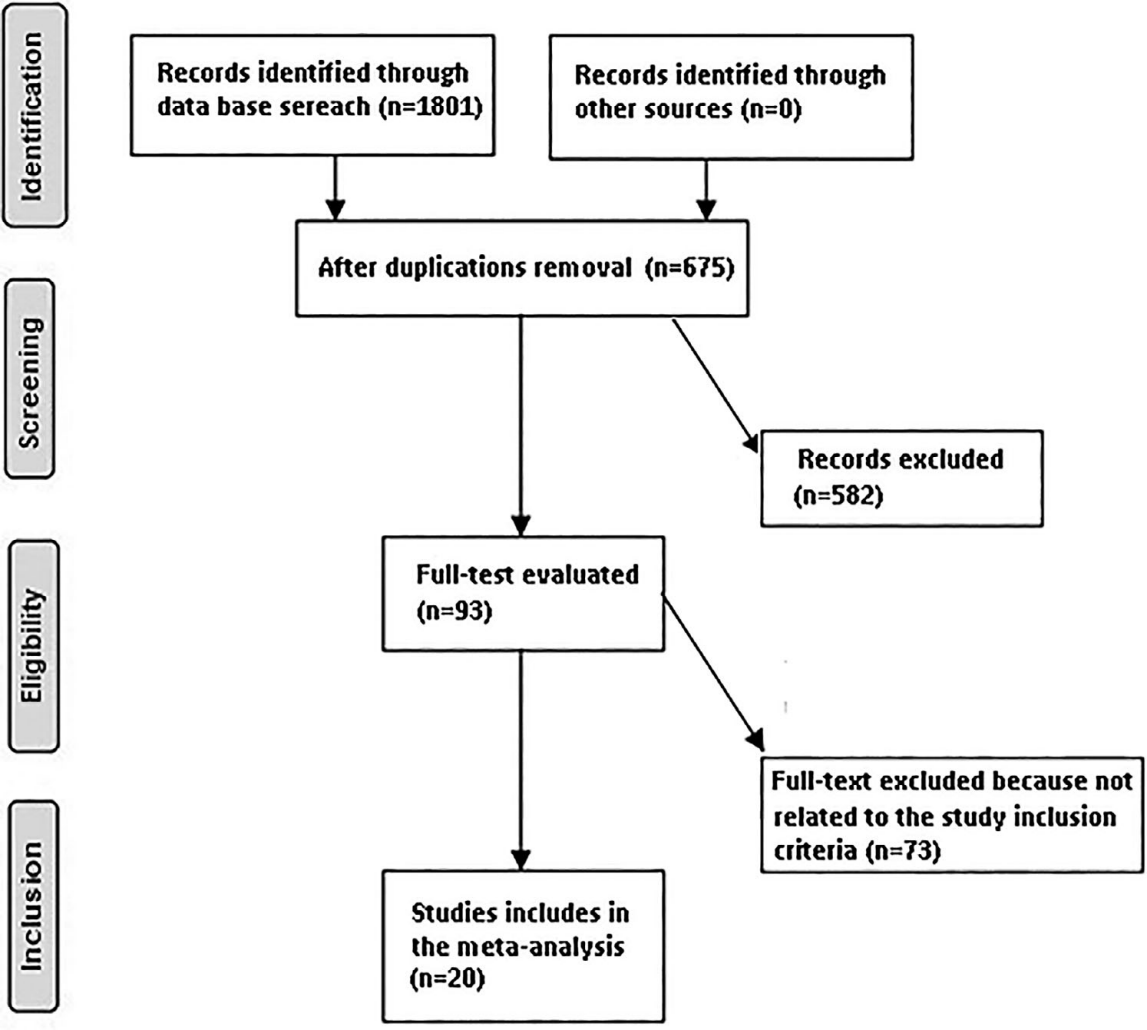
Schematic diagram of the study operation

Only human studies in the English language were considered. Inclusion was not limited by study size or publication type. Publications excluded were review articles and commentary and studies that did not deliver a measure of an association. The articles were integrated into the meta‐analysis when the following inclusion criteria were met: (i) the study was a randomized controlled trial; (ii) the target population was postmenopausal females; (iii) the intervention program was based on osteoporosis drugs' effect on change in lumbar spine bone mineral density; (iv) the study included a comparison between osteoporosis drugs and placebo (Fig. 1).

### Identification

A protocol of search strategies was prepared according to the PICOS principle^32^, and we defined it as follows: P (population): postmenopause females; I (intervention/exposure): osteoporosis drugs effect on change in lumbar spine bone mineral density; C (comparison): osteoporosis drugs compared to placebo; O (outcome): frequency of new vertebral fractures and nonvertebral fractures in postmenopausal females; and S (study design): no restriction^33^.

First, we conducted a systematic search of OVID, Embase, Cochrane Library, PubMed, and Google scholar till May 2020, using a blend of keywords and similar words for an osteoporosis drug, bone mineral density, lumbar spine, vertebral fracture, and nonvertebral fracture as shown in Table 1. All identified studies were pooled in an EndNote file, duplicates were omitted, and the title and abstracts were reviewed to exclude studies that did not report a relationship between the change in lumbar spine bone mineral density and the frequency of new vertebral fractures and nonvertebral fractures in postmenopausal females.

**TABLE 1:** Search strategy for each database

Database	Search strategy
Pubmed	#1 “osteoporosis drug”[MeSH Terms] OR “Bone mineral density”[All Fields] OR “lumbar spine”[All Fields] OR “Vertebral fracture”[All Fields] #2 “nonvertebral fracture”[MeSH Terms] OR “osteoporosis drug”[All Fields] OR “acceptability”[All Fields] OR “Live birth”[All Fields] #3 #1 AND #2
Embase	‘osteoporosis drug’/exp. OR ‘Bone mineral density’/exp. OR ‘lumbar spine’/exp. OR Vertebral fracture #2 ‘nonvertebral fracture’/exp. OR ‘ICBG’/exp. OR ‘acceptability’/exp. OR Live birth #3 #1 AND #2
Cochrane library	(osteoporosis drug):ti,ab,kw (Bone mineral density):ti,ab,kw OR (lumbar spine): ti,ab,kw (Word variations have been searched) #2 (Vertebral fracture):ti,ab,kw OR (nonvertebral fracture):ti,ab,kw OR (acceptability):ti,ab,kw OR (Live birth): ti,ab,kw (Word variations have been searched) #3 #1 AND #2

### Screening

Data were abridged on the following study‐related and subject‐related characteristics onto a standardized form: last name of the primary author, period of study, year of publication, country, region of the studies, and study design; population type, the total number of fractures, demographic data and clinical and treatment characteristics; postoperative risks, qualitative and quantitative method of evaluation, information source, and outcome evaluation; and statistical analysis^34^. When there were different data from one study, we extracted them independently.

The risk of bias in these studies was assessed as follows. Individual studies were evaluated using the quality in prognosis studies tool, which evaluates validity and bias in studies of prognostic factors across six domains: participation, attrition, prognostic factor measurement, confounding measurement and account, outcome measurement and analysis, and reporting^35^. Any inconsistencies were addressed by a re‐evaluation of the original article.

### Eligibility

The main outcome focused on the relationship between the change in lumbar spine bone mineral density and the frequency of new vertebral fractures and nonvertebral fractures in postmenopausal females.

### Inclusion

Sensitivity analyses were limited only to studies reporting the relationship between the change in lumbar spine bone mineral density and the frequency of new vertebral fractures and nonvertebral fractures in postmenopausal females with different osteoporosis drugs compared to placebo. For subcategory and sensitivity analysis, we used comparisons between different osteoporosis drugs compared to placebo.

### Statistical Analysis

The dichotomous method with a random‐effect model or fixed‐effect was used to calculate *OR* and 95% *CI*. The I^2^ index was calculated; the I^2^ index is between 0% and 100%. Values of about 0%, 25%, 50%, and 75% indicate no, low, moderate, and high heterogeneity, respectively^36^. When I^2^ was higher than 50%, we chose the random effect model; when it was lower than 50%, we used the fixed‐effect model. A subcategory analysis was completed by stratifying the original evaluation per outcome categories as described before. In this analysis, a *P*‐value for differences between subcategories of <0.05 was considered statistically significant. Publication bias was evaluated quantitatively using the Egger regression test (publication bias considered present if *P* ≥ 0.05), and qualitatively, by visual examination of funnel plots of the logarithm of *OR*s *vs* their standard errors (SE)^32^. All *P*‐values were two‐tailed. All calculations and graphs were performed using reviewer manager version 5.3 (The Nordic Cochrane Centre, The Cochrane Collaboration, Copenhagen, Denmark).

## Results

### Search

A total of 1801 unique studies were identified, of which 20 studies fulfilled the inclusion criteria and were included in the study^11^, ^12^, ^13^, ^14^, ^15^, ^16^, ^17^, ^18^, ^19^, ^20^, ^21^, ^22^, ^23^, ^24^, ^25^, ^26^, ^27^, ^28^, ^29^, ^30^. Details of included studies are shown in Table 2.

**TABLE 2:** Characteristics of the selected studies for the meta‐analysis

Study	Year	Treatment used	Country	Total	Treatment	Placebo
Harris, 1993^16^	1993	Cyclic etidronate	USA	380	196	184
Liberman, 1995^28^	1995	Alendronate	USA, Belgium, and Israel	881	526	355
Black, 1996^15^	1996	Alendronate	USA	2027	1022	1005
Ettinger, 1999^29^	1999	Raloxifene	North and South America, and Europe	7038	4746	2292
Harris, 1999^19^	1999	Risedronate	USA	1374	696	678
Chesnut III, 2000^20^	2000	Spray salmon calcitonin	USA	1108	838	270
Reginster, 2000^14^	2000	Risedronate	Australia, and Europe	1686	1006	680
Alexandersen, 2001^30^	2001	Iprifravone	Europe	473	234	239
Chesnut III, 2004^18^	2004	Oral ibandronate	USA, and Europe	2929	1954	975
Recker, 2004^17^	2004	Ibandronate	USA, and Europe	2859	1910	949
Meunier, 2004^13^	2004	Strontium ranelate	Australia, and Europe	1442	719	723
Black, 2007^21^	2007	zoledronic acid	USA, New Zealand, and Europe	5675	2822	2853
Cummings, 2008^25^	2008	Tibolone	USA, and Europe	4506	2249	2257
Silverman, 2008^22^	2008	Raloxifene or Bazedoxifene	USA, South Africa, Croatia, Denmark, and Argentina	4991	3735	1256
Cummings, 2009^26^	2009	Denosumab	USA, and Europe	7393	3702	3691
Cummings, 2010^23^	2010	Lafosoxifene	USA, and Europe	8226	5482	2744
Cummings, 2011^24^	2011	Arzoxifene	North and South America, and Europe	9354	4676	4678
Jacques, 2012^12^	2012	Zoledronic Acid	USA	5907	2931	2976
Henriksen, 2016^27^	2016	Oral salmon calcitonin	Brazil, and Europe	4189	2064	2125
Okubo, 2020^11^	2020	Denosumab	Japan	952	472	480
			Total	**73390**	**41980**	**31410**

*Note*: Bold values shown total of the above values.

### Baseline Characteristics

The 20 studies included 73,390 postmenopausal females; of them, a total of 41,980 were treated with osteoporosis drugs, and 31,410 were treated with placebo. All studies were for the determination of the relationship between the change in lumbar spine bone mineral density and the frequency of new vertebral fractures and nonvertebral fractures in postmenopausal females.

Study size ranged from 380 to 9345 subjects at the start of the study with 196 to 5482 treated with osteoporosis drugs. Twenty studies reported data stratified comparison related to vertebral fractures, and 14 studies related to nonvertebral fractures in postmenopausal females.

The extent of the incidence of vertebral fractures and nonvertebral fractures in postmenopausal females was studied. Treatment with osteoporosis drug groups had a significantly lower frequency of new vertebral fractures and nonvertebral fractures compared to placebo in postmenopausal females and this was in all populations studied.

### *Osteoporosis Drugs* vs *Placebo*

**Fig. 2:**
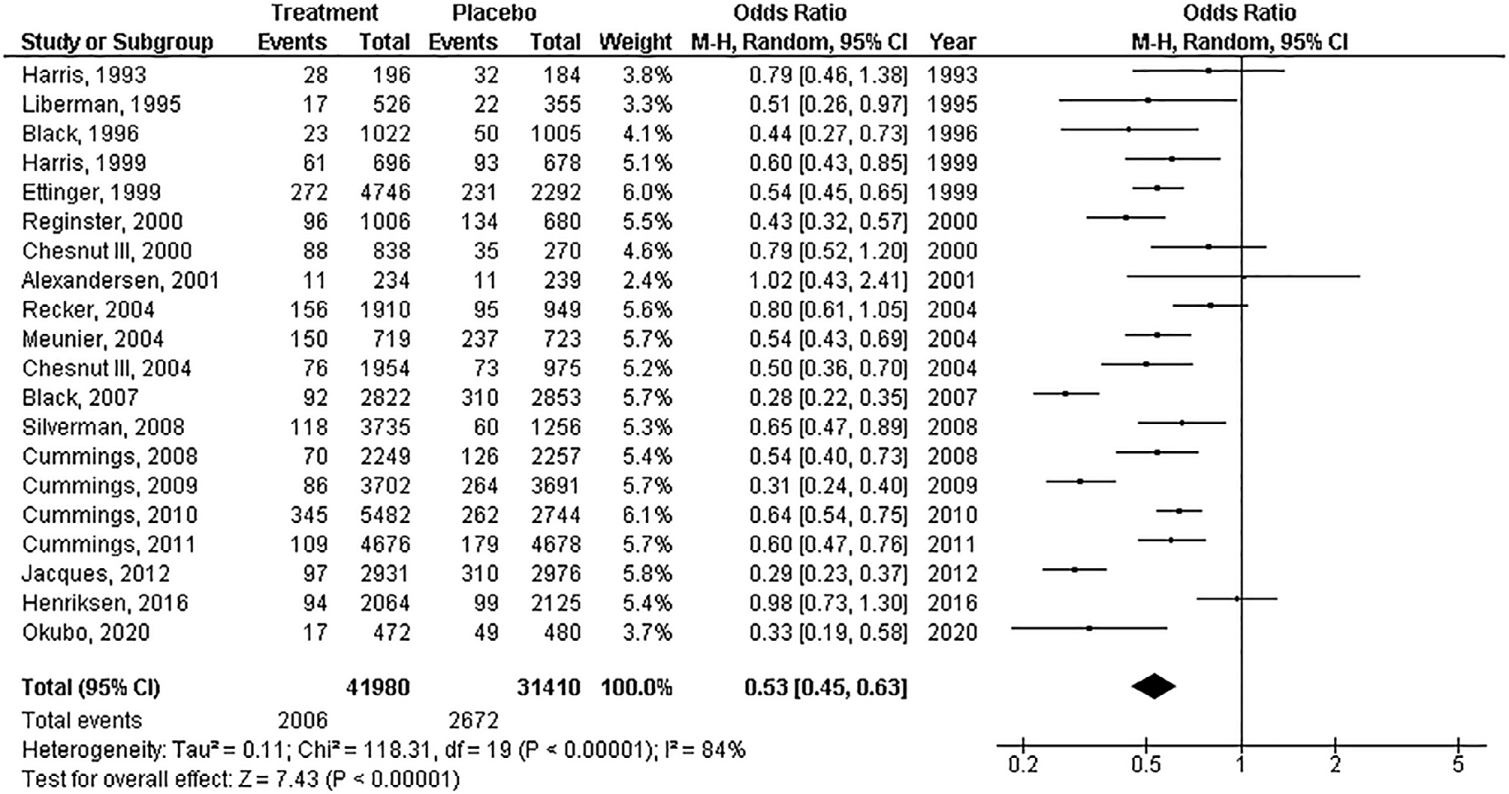
Forest plot of the frequency of new vertebral fractures in treatment with the osteoporosis drugs group compared to the placebo group in postmenopausal females

**Fig. 3:**
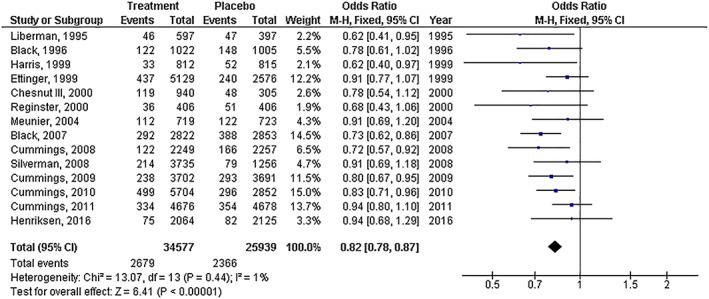
Forest plot of the frequency of new nonvertebral fractures in treatment with the osteoporosis drugs group compared to the placebo group in postmenopausal females

Treatment with osteoporosis drugs had significantly lower frequency of new vertebral fractures (*OR*, 0.53; 95% *CI*, 0.45–0.63, *P*  < 0.001) with high heterogeneity (*I* ^2^ = 84%); and lower nonvertebral fractures (*OR*, 0.82; 95% *CI*, 0.78–0.87, *P*  < 0.001) with no (*I* ^2^ = 1%) compared to placebo in postmenopausal females as shown in Figs 2 and 3.

A stratified analysis of studies that did and did not adjust for the effect of osteoporotic fracture history, gender, and ethnicity on the results was not performed because no studies reported or adjusted for these factors.

### Quality Assessment

**Fig. 4:**
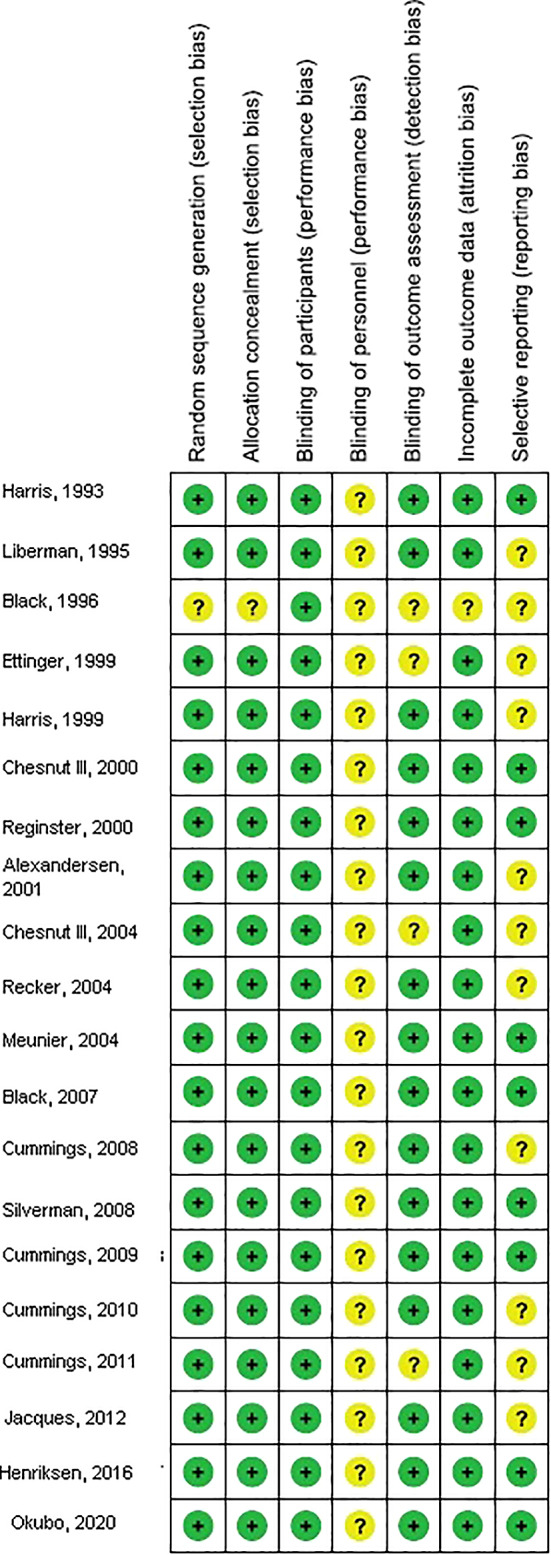
Risk of bias summary

Based on the visual inspection of the funnel plot (Figs S1 and S2, as a visual aid for detecting bias or systematic heterogeneity) as well as on quantitative measurement using the Egger regression test, there was no evidence of publication bias (*P*  = 0.87) as shown in Fig. 4.

## Discussion

### *Osteoporosis Drugs* vs *Placebo*

The relationship between the change in lumbar spine bone mineral density and the frequency of new vertebral fractures and nonvertebral fractures in postmenopausal females was variable in the selected studies. In this meta‐analysis study, based on 20 studies with 73,390 postmenopausal females, a total of 41,980 were treated with osteoporosis drugs and 31,410 with placebo. Treatment with osteoporosis drug groups had a significantly lower frequency of new vertebral fractures and nonvertebral fractures compared to placebo in postmenopausal females. This effect was observed primarily in all subjects^11^, ^12^, ^13^, ^14^, ^15^, ^16^, ^17^, ^18^, ^19^, ^20^, ^21^, ^22^, ^23^, ^24^, ^25^, ^26^, ^27^, ^28^, ^29^, ^30^. This finding suggests that the treatment with osteoporosis drugs had better results in a lower frequency of new vertebral fractures and nonvertebral fractures in postmenopausal females compared to placebo.

The outcomes of this study showed the need for further research on the osteoporosis drugs as a single preventer of the new vertebral fractures and nonvertebral fractures in postmenopausal females to consolidate the finding^11^, ^12^, ^13^, ^14^, ^15^, ^16^, ^17^, ^18^, ^19^, ^20^, ^21^, ^22^, ^23^, ^24^, ^25^, ^26^, ^27^, ^28^, ^29^, ^30^, since the use of osteoporosis drugs in postmenopausal females are controversial. Many studies have been carried out comparing osteoporosis drugs to placebo in postmenopausal females^11^, ^12^, ^13^, ^14^, ^15^, ^16^, ^17^, ^18^, ^19^, ^20^, ^21^, ^22^, ^23^, ^24^, ^25^, ^26^, ^27^, ^28^, ^29^, ^30^.

### Previous Clinical Trial Studies

Previous clinical trial studies of osteoporosis drugs showed that larger intensification in bone mineral density is inclined to have better anti‐fracture efficiency^6^, ^7^. We recommend that the intensification in lumbar spine bone mineral density relates to the inhibition of new fractures under situations where the osteoporosis drug does not disturb the dual‐energy X‐ray absorptiometry quantity. Though, the change in lumbar spine bone mineral density in osteoporosis drug studies presented a significant relationship with the frequency of new fractures irrespective of the modification in the proportion of subjects with predominant vertebral and nonvertebral fracture^11^, ^12^, ^13^, ^14^, ^15^, ^16^, ^17^, ^18^, ^19^, ^20^, ^21^, ^22^, ^23^, ^24^, ^25^, ^26^, ^27^, ^28^, ^29^, ^30^. This outcome showed that the model with the modification more accurately forecasts the frequency of new vertebral fractures and nonvertebral fractures than the model without the modification. Numerous factors could lead to this outcome. First, in a meta‐analysis of cohort studies and the earlier meta‐regression analysis in the placebo group in clinical trials, the frequency of vertebral fractures and nonvertebral fractures has a significant association with the frequency of successive vertebral fractures and nonvertebral fractures^8^, ^10^, ^37^. These outcomes show that the higher the frequency of vertebral fractures and nonvertebral fractures, the higher the frequency of new vertebral fractures and nonvertebral fractures witnessed. So, alterations in the frequency of vertebral fractures and nonvertebral fractures between any study populations ought to be considered when comparing the fracture inhibition effect of a certain drug. Second, the vertebral fracture frequency itself affects bone mineral density quantity. L_1_ is one of the places in which fractures most often happen^38^; one or two fractures in the lumbar spine increase bone mineral density^39^. The International Society of Clinical Densitometry has suggested that anatomically abnormal vertebrae should be excluded from analysis if they are abnormal and non‐quantifiable within the resolution of the system, or if there is more than a 1.0 T‐score variation among the vertebra studied and the adjacent vertebrae^40^. It can be deduced that vertebral fractures and nonvertebral fractures disturb the measurement of lumbar spine bone mineral density, and the frequency of vertebral fractures and nonvertebral fractures decreases the precision of fracture risk forecast by bone mineral density. So, we recommend that the chief factor leading to the outcome of model fitting was the variation in the risk of new vertebral fractures and nonvertebral fractures between the study populations with a different frequency of vertebral fractures and nonvertebral fractures. The current outcomes of the subgroup analysis showed a significant interaction between the proportion of subjects with predominant vertebral fractures and nonvertebral fractures and the percentage variation in lumbar spine bone mineral density from baseline at 3 years^41^, ^42^, ^43^, ^44^. The degree of frequency of vertebral fractures and nonvertebral fractures in the population must be considered when the correlation between variation in lumbar spine bone mineral density and frequency of vertebral fractures and nonvertebral fractures is observed.

### Recommendations

From the present study, Treatment with osteoporosis drugs had a significantly lower frequency of new vertebral fractures and nonvertebral fractures compared to placebo in postmenopausal females^11^, ^12^, ^13^, ^14^, ^15^, ^16^, ^17^, ^18^, ^19^, ^20^, ^21^, ^22^, ^23^, ^24^, ^25^, ^26^, ^27^, ^28^, ^29^, ^30^. These outcomes have vital benefits in postmenopausal females^11^, ^12^, ^13^, ^14^, ^15^, ^16^, ^17^, ^18^, ^19^, ^20^, ^21^, ^22^, ^23^, ^24^, ^25^, ^26^, ^27^, ^28^, ^29^, ^30^.

This relationship forces us to recommend osteoporosis drugs in postmenopausal females to avoid any possible new fractures. A cost‐effective study is recommended for better results.

Our meta‐analysis study could not answer whether the effect of osteoporotic fracture history, gender, and ethnicity are associated with different results since most of the studies did not adjust for these factors. Larger prospective studies are recommended to confirm these findings and adjust for the effect of osteoporotic fracture history, gender, and ethnicity.

## Limitations

First, the analysis was not completed at the patient level but was instead based on summary data. Second, data from randomized clinical trials were used in this study. The features of subjects in clinical trials of a new treatment may have influenced the generalizability of this study outcome. Third, numerous latest clinical trials for osteoporosis drugs such as romosozumab and odanacatib, which showed radical intensifications in bone mineral density, were not included in this study due to their short study period or hidden study outcomes. Further studies are needed to show why these big variations in bone mineral density in a short study period occurred.

### Conclusions

Treatment with osteoporosis drugs had a significantly lower frequency of new vertebral fractures and nonvertebral fractures compared to placebo in postmenopausal females. This relationship forces us to recommend osteoporosis drugs in postmenopausal females to avoid any possible new fractures. However, the degree of frequency of vertebral fractures and nonvertebral fractures in the population should be considered when the association between variation in lumbar spine bone mineral density and frequency of vertebral fractures and nonvertebral fractures is inspected. Also, cost‐effective studies are needed.

## Declarations

### Ethics approval and consent to participate

Not applicable.

### Consent for publication

Not applicable.

### Availability of data and materials

The datasets analyzed during the current study are available from the corresponding author on reasonable request.

The authors declare that they have no competing interests.

### Authors' Contributions

Conception and design: LC and MA. Administrative support: All authors. Provision of study materials or subjects: All authors. Collection and assembly of data: LC, JC, LZ. Data analysis and interpretation: All authors. Manuscript writing: All authors. Final approval of manuscript: All authors. All authors have read and approved the manuscript.

### IRB Approval

Not required for this study.

## Supporting information


**Fig. S1** Funnel plot Vertebral fracture

**Fig. S2** Funnel plot Nonvertebral fracture

**Table S1** Filled PRISMA Checklist
