# Supplementary figures and images for: Relationship between Change in Bone Mineral Density of Lumbar Spine and Risk of New Vertebral and Nonvertebral Fractures: A Meta‐Analysis
Source: Orthop Surg. 2022 Jan 4;14(2):199–206. doi: 10.1111/os.13184 (PMC8867431; doi:10.1111/os.13184)

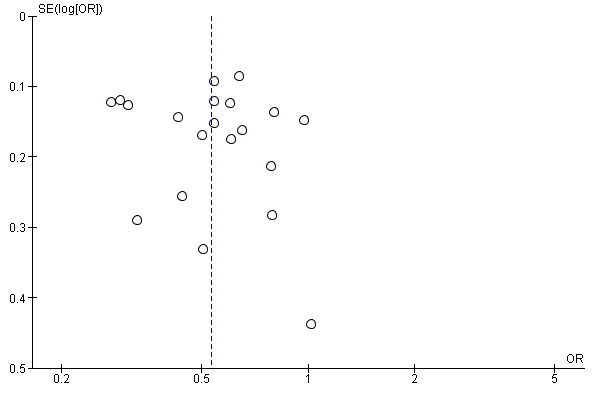

Supplement: Supplementary file 1 — Fig. S1 Funnel plot Vertebral fracture [file OS-14-199-s001.jpg]

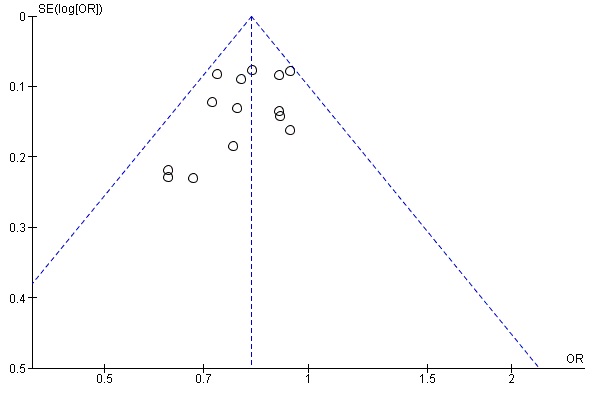

Supplement: Supplementary file 2 — Fig. S2 Funnel plot Nonvertebral fracture [file OS-14-199-s002.jpg]
